# The Role of Ferroptosis in Bronchoalveolar Epithelial Cell Injury Induced by Cigarette Smoke Extract

**DOI:** 10.3389/fphys.2021.751206

**Published:** 2021-09-29

**Authors:** Ningfang Lian, Qiaoxian Zhang, Jia Chen, Mengxue Chen, Jiefeng Huang, Qichang Lin

**Affiliations:** Department of Pulmonary and Critical Care Medicine, The First Affiliated Hospital of Fujian Medical University, Institute of Respiratory Disease, Fujian Medical University, Fuzhou, China

**Keywords:** lung injury, ferroptosis, cigarette smoke extract, RNA sequencing, bronchoalveolar epithelial cell

## Abstract

**Background:** Cigarette smoking is a major risk factor for bronchoalveolar epithelial cell (BAEC) injury. Understanding the relevant pathogenesis is important for the treatment of cigarette smoke–related chronic airway diseases such as chronic obstructive pulmonary disease.

**Methods:** In this study, BAECs were cultured in 5% cigarette smoke extract (CSE) or regular culture medium for 24 h. Differentially expressed genes (DEGs) were detected by next-generation RNA sequencing (RNA-seq) and validated by quantitative reverse transcription polymerase chain reaction (qRT-PCR). Bioinformatic analysis was performed on DEGs. Co-treated BAECs with 5% CSE and the ferroptosis inhibitor, ferrostatin-1 was applied to observe the role of ferroptosis.

**Results:** In the CSE group, 210 upregulated genes and 159 downregulated genes were identified compared with the control group. Gene Ontology and Kyoto Encyclopedia of Genes and Genomes (KEGG) analyses showed that the DEGs were related to oxidative stress and ferroptosis. Ferroptosis-related genes were further verified by qRT-PCR. The mRNA level of GPX4 decreased; the mRNA levels of ACSL4, FTH1 and SLC7A11 increased (*p* < 0.05). Pretreatment with the ferroptosis inhibitor ferrostatin-1 mitigated CSE-induced ROS accumulation and inflammatory mediator expression in BAECs (*p* < 0.05).

**Conclusion:** CSE treatment altered ferroptosis-related gene expression patterns in cultured BAECs. Inhibition of ferroptosis reduced the inflammatory response of CSE-treated BAECs. These data provide a better understanding of the underlying molecular mechanisms of CSE-related lung injury.

## Introduction

Chronic obstructive pulmonary disease (COPD) is a disease with high morbidity and high mortality (Halpin et al., 2020). COPD is characterized by airway inflammation, and while traditional treatment methods such as bronchodilators and glucocorticoids alleviate symptoms, they cannot prevent disease progression (Nici et al., 2020; Vogelmeier et al., 2020; Baha and Kokturk, 2021). Therefore, it is important to find new targets for COPD prevention and treatment.

Exposure to the harmful components in tobacco is a major risk factor for COPD and directly injures bronchoalveolar epithelial cells. Although previous studies have shown that cigarette smoke extract (CSE) causes airway inflammation and apoptosis of alveolar epithelial cells (Cui et al., 2018; Sun et al., 2019). There are currently no specific treatments to reverse these injuries in clinical practice. In the past decade, advances in next-generation sequencing have provided us with a better understanding of the pathogenesis of many diseases. However, relatively a few studies have focused on gene expression changes induced by CSE in alveolar epithelial cells.

Ferroptosis is an atypical form of regulated cell death, characterized by iron-dependent accumulation of lethal lipid reactive oxygen species (Li et al., 2020). Ferroptosis is distinct from apoptosis, pyroptosis, and necroptosis. It has been reported in a large number of studies in recent years and is associated with multiple pathological processes such as cancer, stroke and obstructive sleep apnea (Chen et al., 2020; Zhou et al., 2020; Xu et al., 2021). In previous study, RNA sequencing (RNA-seq) revealed that several messenger RNAs (mRNAs) related to ferroptosis were enriched in vascular smooth muscle cells pretreated with CSE (Sampilvanjil et al., 2020). However, the role of ferroptosis in CSE-related alveolar epithelial cell injury is not well understood.

The aim of this study was to explore the gene expression profile of CSE-treated alveolar epithelial cells *via* RNA-seq and perform bioinformatic analysis to clarify the functions of differentially expressed genes (DEGs). A secondary aim was to explore the role of ferroptosis and provide new insights for the treatment of CSE-related airway epithelial lesions.

## Materials and Methods

### Cigarette Smoke Extract Preparation

Cigarette smoke extract preparation followed a previously reported method with some modifications (Higashi et al., 2014, 2016). One piece of cigarette (Septwolves, Longyan Cigarette Factory, Fujian, China) was burned in a 500 ml bottle with a modified 50-ml syringe-driven apparatus. The smoke was dissolved in 20 ml of RPMI-1640 medium. To remove large particles and bacteria, the resulting solution was filtered through a 0.22-μm pore-size filter and adjusted to a pH of 7.4. The solution was identified as 100% CSE. Concentrations of 5% CSE were obtained by diluting 100% CSE in RPMI-1640 complete medium. Within 30 min of preparation, 5% CSE was used for subsequent experiments.

### Establishment of the Cell Model

Bronchoalveolar epithelial cells (BAECs) were obtained from Biyuntian Biological Technology Co., Ltd., (Shanghai, China). Complete medium was made from RPMI-1640 (HyClone Laboratory Inc., United States) supplemented with 10% fetal bovine serum (Gibco Life Technologies Inc., United States). Cells were cultured in the incubator at 37°C (Thermo Fisher Scientific, Waltham, MA, United States) with a humidified atmosphere of a 5% CO2. In CSE group, once BAECs cells reached 70–80% confluency, replaced complete medium with 5% CSE. CSE exposure was applied for 24 h. In CSE + ferrostatin-1 group, co-treated BAECs with 5% CSE and 1 μM ferroptosis inhibitor, ferrostatin-1 was applied for 24 h.

### RNA Isolation, Library Preparation, and Illumina Sequencing

After extracting total RNA from BAECs by TRIzol Reagent (Invitrogen, United States), the quality and quantity of the total RNA were quantified *via* DNA/RNA concentration assay (Pharmathea Inc., China). Poly-T oligo-attached magnetic beads were then used to purify the mRNA. First-strand cDNA and second-strand cDNA was synthesized with random Primers and dUTP respectively. End-repair and ligation with “A-tailing” base adaptors were performed in all synthetic cDNA. After that, polymerase chain reaction (PCR) was used to amplify suitable fragments and construct the cDNA library. The quantity and quality of the sample library were verified by qPCR quantification methods and Agilent 2100 Bioanalyzer (Agilent, United States). The libraries were sequenced by NovaSeq 6000 (Illumina, United States).

### Next-Generation RNA Sequencing Data Processing

After quality control and removal of ribosomal RNA, the raw sequence data were detected with adaptor sequences, removed street sequences and short fragments in Read. The trimmed data thus obtained were compared to the reference genome. Fragments per kilobase of exons per million fragments mapped (FRKM) were used to calculate transcript levels. The genes with a FRKM > 0.5 were included for follow-up analysis. Genes with *p*-value < 0.05 and a CSE vs. control fold change > 1.50 (or <1/1.5) were defined as DEGs.

### Bioinformatic Analysis

Volcano graph, scatter plots, and hierarchical clustering were used to visualize the differential gene expression profiles. Functional enrichment of DEGs based on the DAVID database was performed *via* Gene Ontology (GO) enrichment analysis^1^. Signal pathways involving DEGs were obtained *via* Kyoto Encyclopedia of Genes and Genomes (KEGG) pathway enrichment analysis^2^. Significant GO terms and pathways were identified *via* Fisher’s exact test. A *p* value < 0.05 was regarded as significant.

### Quantitative Reverse Transcription Polymerase Chain Reaction

Total RNA was reverse transcribed into cDNA by PrimeScript RT reagent Kit (Takara Bio Inc., Japan). To determine the mRNA expression levels of *Glutathione peroxidase 4 (GPX4), acyl-CoA synthetase long-chain family member 4 (ACSL4), Recombinant Solute Carrier Family 7, Member 11 (SLC7A11), ferritin heavy chain 1 (FTH1), interleukin-6 (IL6*) and *tumor necrosis factor-alpha* (*TNF-*α), the SYBR Green PCR Master Mix (Takara Bio Inc., Japan) was used and RT-qPCR was performed on an ABI 7500 thermocycler (Applied Biosystems, Foster City, CA, United States). Relevant primers are listed in Table 1. β-Actin was used as an internal control. Fold changes were calculated by the 2^–ΔΔCT^ method.

**TABLE 1:** List of primer sequence.

Genes	Primer sequence
FTH1	F: TCCTACGTTTACCTGTCCATGT
	R: GTTTGTGCAGTTCCAGTAGTGA
GPX4	F: CCGCTGTGGAAGTGGATGAAGATC
	R: CTTGTCGATGAGGAACTGTGGAG
SLC7A11	F: ATGCAGTGGCAGTGACCTTT
	R: GGCAACAAAGATCGGAACTG
ACSL4	F: TCTGCTTCTGCTGCCCAATT
	R: CGCCTTCTTGCCAGTCTTTT
IL6	F: GGTGTTGCCTGCTGCCTTCC
	R: GTTCTGAAGAGGTGAGTGGCTGTC
TNFα	F: TGGCGTGGAGCTGAGAGATAACC
	R: CGATGCGGCTGATGGTGTGG
Beta-actin	F: TGGCACCCAGCACAATGAA
	R: CTAAGTCATAGTCCGCCTAGAAGCA

### Cell Viability Test

A Cell Counting Kit-8 assay (Beyotime Bio Inc., China) was used to detect cell viability. Five thousand cells per well were added to a 96-well plate, with five replicate wells in each group. phosphate-buffered saline (PBS) was added to surrounding wells to reduce errors. After 12 h, 5% CSE was added in CSE group. After modeling, 10 μL CCK-8 solution was added to each well and the 96-well plate was incubated at 37°C for 1 h. The absorbance at 450 nm was detected with a microplate reader. Cell viability was calculated as (experimental group-blank control) / (negative control-blank control) × 100%.

### Apoptosis Assay

Cells were double stained with Annexin V-fluorescein isothiocyanate (FITC) and propidium iodide (PI) (Beyotime Bio Inc., China). The cell apoptosis rate was then assessed by flow cytometry analysis. In brief, 1 × 10^5^ cells were collected per well, with three replicate wells in each group. After washing in PBS, cells were resuspended in 200 μl binding buffer, mixed with 10 μl of PI and 5 μl of Annexin V-FITC. The suspensions were analyzed by a C6 flow cytometer (Becton Dickinson, United States).

### Intracellular Reactive Oxygen Species

Intracellular reactive oxygen species (ROS) were detected by the Reactive Oxygen Species Assay Kit (Beyotime Bio Inc., China). The DCFH-DA fluorescent probe and serum-free culture medium were diluted at a ratio of 1:1,000. One milliliter of diluted DCFH-DA solution was added to one well of the 12-well plates, with three replicate wells in each group. After incubation for 20 min in a 37°C incubator, cells were collected for flow cytometry analysis.

### Statistical Analysis

When the data were normally distributed, the mean ± standard deviation was used; otherwise, the interquartile range was used. An independent *t* test was used to compare the mean of continuous variables in normally distributed data; the Mann–Whitney test was used to compare non-normally distributed data. A *p* value < 0.05 was considered statistically significant. Statistical analysis was performed by Graphpad Prism 7.0 (GraphPad Software Inc., United States).

## Results

### Effects of Cigarette Smoke Extract on Cell Viability in Bronchoalveolar Epithelial Cells

**FIGURE 1:**
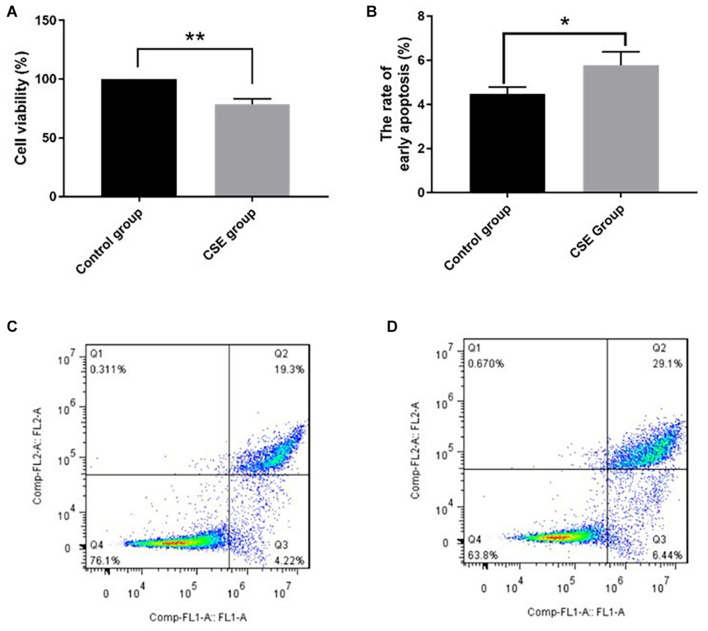
CSE-induced BAEC injury (*n* = 3). **(A)** CSE reduced cell viability of BAECs; **(B)** CSE increased the early apoptosis rate of BAECs; **(C)** apoptosis rate in the control group; **(D)** apoptosis rate in the CSE group. CSE, cigarette smoke extract; BAEC, bronchoalveolar epithelial cells. *p < 0.05; **p < 0.01.

After 24-h exposure to 5% CSE, BAEC viability decreased significantly compared with the control group, (78.7 ± 4.8)% vs. (100.0 ± 0.0)%, *p* < 0.05. The rate of early apoptotic BAECs increased in the CSE group compared with the control group, (5.78 ± 0.61)% vs. (4.47 ± 0.32)%, *p* < 0.05. These data support our CSE-induced injury model in BAECs, as shown in Figure 1.

### Identification of Differentially Expressed Genes

**FIGURE 2:**
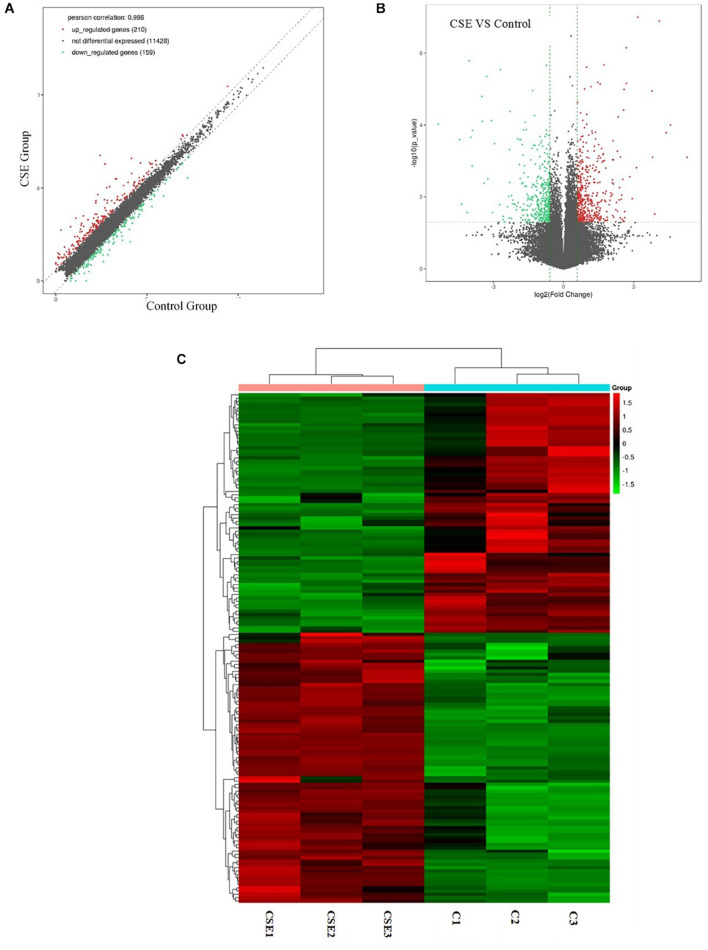
Next-generation RNA sequencing data showing the differentially expressed genes in CSE group and control group of BAECs (*n* = 3). **(A)** Differentially expressed genes in the CSE and control groups. **(B)** Volcano plot showing differentially expressed genes in the CSE and control groups. Genes that were differentially expressed with a fold change > 1.5 and *p* < 0.05 are marked in color. **(C)** Heat map of differentially expressed genes in the CSE and control groups. CSE, cigarette smoke extract; BAEC, bronchoalveolar epithelial cells.

Differentially expressed genes were identified in CSE-treated BAECs by RNA-seq. With a fold change cutoff of 1.5 (or less than 1/1.5) and *p* < 0.05, 369 dysregulated genes (210 upregulated genes and 159 downregulated genes) were identified in the BAECs exposed to 5% CSE compared with controls. To visualize the DEGs between the CSE group and the control group, volcano plots were performed by fold change values and *p* values; Gene expression differences are also presented as scatter plots (Figures 2A,B). Hierarchical clustering was conducted to predict the function of DEGs (Figure 2C). *SERPINB2, SHISA2, NQO1, PRDM2, CYP1B1, STC2, AHRR, TRIM16L, EREG and SLC7A11* were the top 10 upregulated genes, as shown in Table 2. *JAG1, S100A2, NOTCH3, RPL36A-HNRNPH2, MEGF6, SULF1, EPPK1, SLIT3, CD14 and CTGF* were the top 10 downregulated genes, as shown in Table 3.

**TABLE 2:** List of top ten upregulated DEGs in CSE group compared to control group.

Gene name	Description	Chromosome	Fold change	*p* value
SERPINB2	Serpin Family B Member 2	18	5.40	0.001
SHISA2	Shisa Family Member 2	13	4.28	0.001
NQO1	NAD(P)H Quinone Dehydrogenase 1	16	3.21	0.0002
PRDM2	PR/SET Domain 2	1	3.19	0.0001
CYP1B1	Cytochrome P450 Family 1 Subfamily B Member 1	2	3.06	0.0004
STC2	Stanniocalcin 2	5	2.84	0.023
AHRR	Aryl-Hydrocarbon Receptor Repressor	5	2.68	0.011
TRIM16L	Tripartite Motif Containing 16 Like	17	2.63	0.001
EREG	Epiregulin	4	2.52	< 0.001
SLC7A11	Solute Carrier Family 7 Member 11	4	2.50	0.0004

*DEP, differentially expressed genes; CSE, Cigarette smoke extract.*

**TABLE 3:** List of top ten downregulated DEGs in CSE group compared to control group.

Gene name	Description	Chromosome	Fold change	*p* value
JAG1	Jagged Canonical Notch Ligand 1	20	0.51	0.003
S100A2	S100 Calcium Binding Protein A2	1	0.49	0.008
NOTCH3	Notch Receptor 3	19	0.49	0.031
RPL36A-HNRNPH2	RPL36A-HNRNPH2 Readthrough	X	0.47	0.042
MEGF6	Multiple EGF Like Domains 6	1	0.46	0.008
SULF1	Sulfatase 1	8	0.46	0.002
EPPK1	Epiplakin 1	8	0.45	0.009
SLIT3	Slit Guidance Ligand 3	5	0.44	0.001
CD14	CD14 Molecule	9	0.43	0.0002
CTGF	Cellular Communication Network Factor 2	6	0.42	0.005

*DEP, differentially expressed genes; CSE, Cigarette smoke extract.*

### Gene Ontology Analysis

**FIGURE 3:**
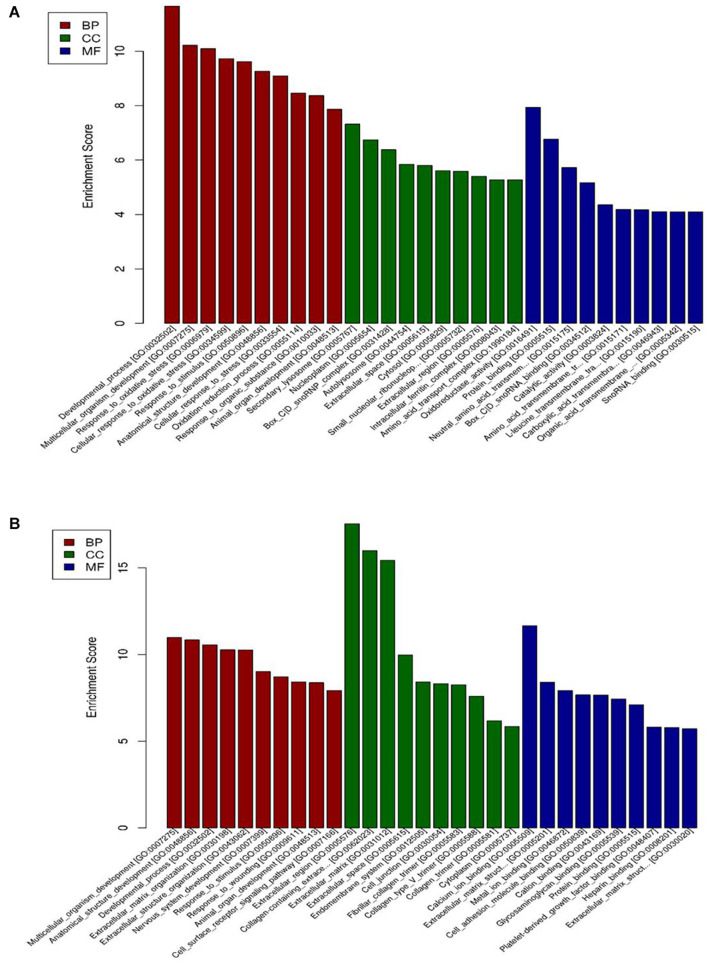
GO enrichment-based clustering analysis of the differentially expressed genes in BAECs (*n* = 3). **(A)** The top 10 GO terms associated with upregulated genes in the CSE group compared with the control group; **(B)** the top 10 GO terms associated with downregulated genes in the CSE group compared with the control group. CSE, cigarette smoke extract; BAEC, bronchoalveolar epithelial cells; CC, Cellular component analysis; BP, Biological process analysis; MF, Molecular function analysis.

The DEGs were classified by the GO database on the basis of the categories of biological process (BP), cellular component (CC), and molecular function (MF). The top ten GO terms for upregulated genes and downregulated genes are shown in Figure 3. The dysregulated genes were enriched in BP terms including response to oxidative stress and developmental process, CC terms including nucleoplasm and extracellular region, and MF terms including metal ion binding, oxidoreductase activity, and protein binding.

### Kyoto Encyclopedia of Genes and Genomes Analysis

**FIGURE 4:**
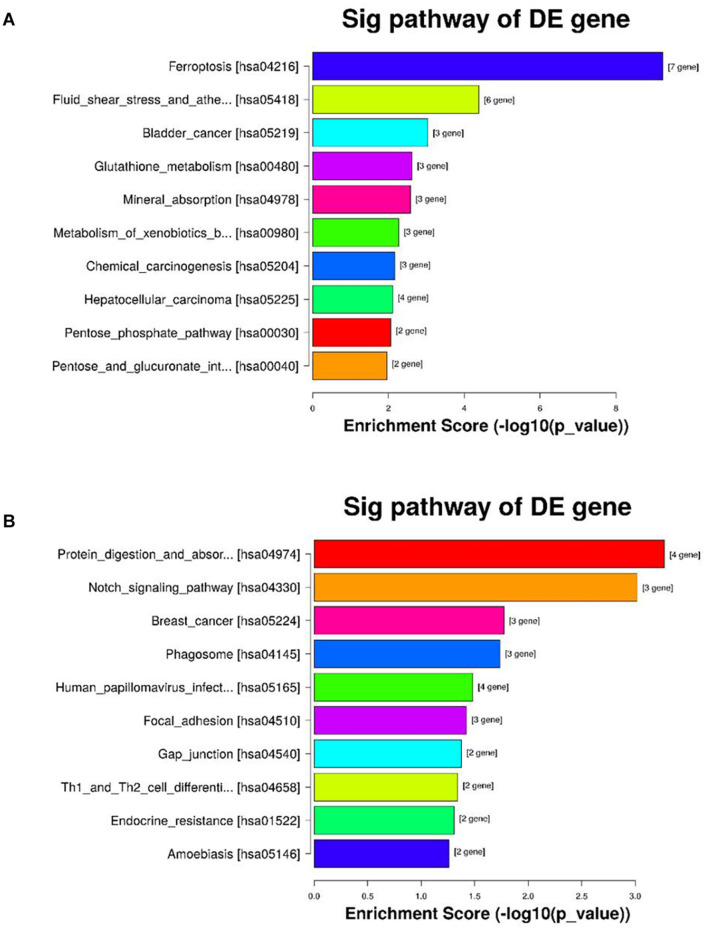
KEGG pathway enrichment of the differentially expressed genes in the CSE and control groups of BAEC (*n* = 3). **(A)** The top 10 KEGG pathways associated with upregulated genes in the CSE group compared with the control group; **(B)** the top 10 KEGG pathways associated with downregulated genes in the CSE group compared with the control group. KEGG, Kyoto Encyclopedia of Genes and Genomes; CSE, cigarette smoke extract; BAEC, bronchoalveolar epithelial cells.

Kyoto encyclopedia of genes and genomes pathway analysis was conducted to find potential biological functions of the significant DEGs. Twenty signaling pathways, including ferroptosis, glutathione metabolism and cancer, were associated with the upregulated DEGs and 12 signaling pathways, including protein digestion and absorption, the Notch signaling pathway, Th1 and Th2 cell differentiation and the Mitogen-activated protein kinase (MAPK) signaling pathway, were associated with the downregulated DEGs. The top 10 enriched pathways sorted by enrichment score are showed in Figure 4.

### Validation of Cigarette Smoke Extract-Induced Ferroptosis Gene Expression by Quantitative Reverse Transcription Polymerase Chain Reaction

**FIGURE 5:**
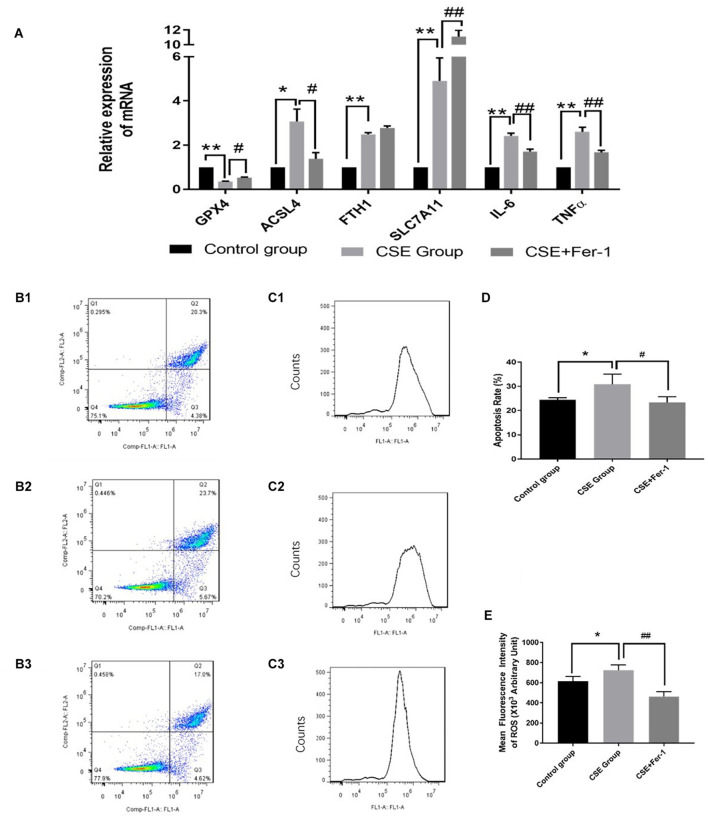
Effects of ferrostatin-1 on mRNA expression levels, apoptosis rates and ROS levels (*n* = 3). **(A)** *GPX4, ACSL4, FTH1, SLC7A11, IL6* and *TNF-*α mRNA levels in control group, CSE group and CSE + ferrostatin-1 group of BAECs. **(B1–3,D)** The apoptosis rate in control group, CSE group and CSE + ferrostatin-1 group of BAECs. **(C1–3,E)** ROS levels in control group, CSE group and CSE + ferrostatin-1 group of BAECs. CSE, cigarette smoke extract; BAEC, bronchoalveolar epithelial cells. **p < 0.01 compared with the control group; *p < 0.05 compared with the control group; ##*p* < 0.01 compared with the CSE group; #*p* < 0.05 compared with the CSE group.

Quantitative reverse transcription polymerase chain reaction (qRT-PCR) was performed to verify the differential expression of ferroptosis genes in the CSE group compared with the control group. Compared with the control group, *ACSL4, FTH1*, and *SLC7A11* mRNA levels were significantly upregulated in CSE group, while *GPX4* mRNA expression was significantly downregulated in CSE group, *p* < 0.05 (Figure 5A).

### Ferrostatin-1 Alleviated Cigarette Smoke Extract-Induced Inflammation and Ferroptosis in Bronchoalveolar Epithelial Cells

TNF-α and IL6 are biomarkers of inflammation. The qRT-PCR results suggested that CSE treatment increased mRNA levels of TNF-α and IL6, which reflected CSE treatment induced inflammation in BAECs. Therefore, we co-treated BEACs with CSE and the ferroptosis inhibitor, ferrostatin-1. Compared with CSE treatment alone, incubation with CSE + ferrostatin-1 resulted in decreased mRNA expression of TNF-α and IL6, *p* < 0.05 (Figure 5A). Ferrostatin-1 also changed the mRNA levels of *GPX4* and *ACSL4* in CSE treated BAECs, *p* < 0.05 (Figure 5A).

The results of flow cytometry analysis suggested that CSE treatment increased the apoptosis rate of BAECs, while co-treated BEACs with CSE and ferrostatin-1 alleviated apoptosis of BAECs (Figures 5B,D). The DCFH-DA fluorescent probe assay showed increased ROS levels in CSE-treated BAECs. Compared with the CSE group, ROS levels were lower in the CSE + ferrostatin-1-treated group (Figures 5C,E).

## Discussion

In this study, we found 210 upregulated genes and 159 downregulated genes in CSE-treated BAECs compared with controls. Gene ontology and KEGG analysis revealed that the DEGs were enriched for the ferroptosis pathway. Moreover, ferrostatin-1, a ferroptosis inhibitor, abrogated part of the CSE-induced gene expression, apoptosis and ROS changes in BAECs.

RNA-seq is an efficient high-throughput technology that has uncovered the potential molecular mechanisms of various diseases (Morrow et al., 2019). However, few RNA-seq studies have focused on CSE-related BAEC injury. In this study, we made a BAECs injury model *via* CSE and searched for DEPs using RNA-seq technique. In a subsequent step, GO enrichment analysis showed that the upregulated DEGs of the CSE-treated BAECs were involved in oxidative stress pathways and were associated with molecular functions such as protein binding, amino acid, and leucine transport. Oxidative stress plays an important role in cigarette smoke–related COPD (Barnes, 2017, 2020; Zhang et al., 2020). The differential gene expression uncovered in this study may provide new ideas for future studies on the etiology and potential treatment of COPD.

In previous studies, GPX4 expression was downregulated in mice with COPD (Yoshida et al., 2019). GPX4 is a key regulator of ferroptosis (Seibt et al., 2019). In this study, we confirmed downregulation of *GPX4* mRNA, and also found that *ACSL4, FTH1*, and *SLC7A11* mRNA expression was significantly increased in CSE-treated BAECs compared with controls. These data further confirm that cigarette smoke likely results in ferroptosis in BAECs. The consumption or inactivation of *GPX4* leads to lipid peroxide accumulation in cells and resulting ferroptosis (Imai et al., 2017). The co-culture of ferrostatin-1 may affect the mRNA levels of *GPX4* and *ACSL4* by inhibiting lipid peroxidation, but did not affect the expression level of *FTH1*. *SLC7A11* is a glutamate-cystine antiporter in the phospholipid bilayer (Okazaki et al., 2019; Koppula et al., 2020). *SLC7A11* may indirectly reduce the effect of GPX4 by reducing the transport of glutathione, which leads to intracellular lipid peroxidation and ferroptosis. In this study, the further increased expression of *SLC7A11* after ferrostatin-1 co-culture was confusing. Ferrostatin-1 may not affect the transport of glutathione, we will explore the mechanisms in future studies.

In addition to oxidative stress, inflammation also plays an important role in the pathogenesis of COPD (Barnes, 2016; Higham et al., 2019). Ferroptosis induces bronchial epithelial cells to release pro-inflammatory cytokines, which forms an inflammatory cascade that leads to COPD-related airway remodeling and emphysema. Ferrostatin-1 is a highly effective non-apoptotic, erastin-induced ferroptosis inhibitor (Bai et al., 2020; Liu et al., 2020). Previous *in vivo* and *in vitro* studies have confirmed that ferrostatin-1 can reduce lung injury caused by lipopolysaccharides (Liu et al., 2020). In our study, CSE increased expression of inflammatory mediators *IL-6* and *TNF-*α in BAECs. In contrast, ferrostatin-1 pretreatment reduced expression of inflammatory mediators, apoptosis rate and ROS in our cultured BAECs. These data suggest that ferroptosis is, at least partially, responsible for CSE-induced BAEC injury in this study.

There are some limitations to this study. First, this is an *in vitro* cell-based study, and the results have not been validated in animal models or human patients. There may be differences in the results of *in vitro* and *in vivo* experiments, which may affect the reproducibility of our conclusions. Secondly, because of funding limitations, the number of samples included in the RNA-seq experiment was relatively small.

In conclusion, our data indicate that CSE exposure alters the gene expression profile of cultured BAECs and that differentially expressed genes are enriched for the ferroptosis pathway. The ferroptosis inhibitor ferrostatin-1 inhibited ROS accumulation and inflammatory mediator expression in CSE-treated BAECs. These results suggest that cigarette smoking may induce ferroptosis in airway epithelium, which could contribute to CSE-related airway dysfunction.

## Data Availability Statement

BioProject’s metadata is available at https://www.ncbi.nlm.nih.gov/bioproject/PRJNA761787.

## Author Contributions

NL, QZ, and QL: conception and design. NL, QZ, and MC: collection and assembly of data. NL and JC: data analysis and interpretation. NL and JH: manuscript revision. All authors wrote the manuscript and approved the final version of the manuscript.

## Conflict of Interest

The authors declare that the research was conducted in the absence of any commercial or financial relationships that could be construed as a potential conflict of interest.

## Publisher’s Note

All claims expressed in this article are solely those of the authors and do not necessarily represent those of their affiliated organizations, or those of the publisher, the editors and the reviewers. Any product that may be evaluated in this article, or claim that may be made by its manufacturer, is not guaranteed or endorsed by the publisher.

## Footnotes

1
http://www.geneontology.org

2
http://www.genome.jp/kegg
